# Osteoid osteoma of the femoral head treated by radiofrequency ablation: a case report

**DOI:** 10.1186/1752-1947-5-115

**Published:** 2011-03-24

**Authors:** Koyeli M Mahata, Shyam KN Keshava, Korula M Jacob

**Affiliations:** 1 Department of Radiodiagnosis, Christian Medical College and Hospital, Vellore, Tamil Nadu, India; 2 Department of Orthopaedics Unit II, Christian Medical College and Hospital, Vellore, Tamil Nadu, India

## Abstract

**Introduction:**

We present a case report highlighting the unusual location and atypical imaging characteristics of an osteoid osteoma in the juxta-articular region of the femoral head, and treatment of the condition with radiofrequency ablation. This treatment option is low in both risk and morbidity and is therefore the best option in lesions that are difficult to access surgically because of the risks involved.

**Case presentation:**

A 40-year-old Indian man from West Bengal presented to our facility with a history of progressively severe left hip pain of insidious onset, requiring analgesics. Imaging with plain radiographs, computed tomography and magnetic resonance imaging confirmed findings of osteoid osteoma in a subarticular location in the femoral head, although imaging features were atypical due to the intra-articular subchondral location.

**Conclusion:**

Radiofrequency ablation is a newer treatment modality for osteoid osteoma that, being minimally invasive, offers comparable results to surgery with a significantly lower morbidity. To the best of our knowledge, treatment of osteoid osteoma in the foveal region of the femoral head with radiofrequency ablation has not been reported to date. We wish to highlight the successful outcome in our index case using this technique.

## Introduction

Osteoid osteomas represent 12% of benign bone tumors and were first described by Jaffe in 1935 [1]. They are twice as common in males; 90% occurring between 5 and 30 years of age [2]. In over 50% of cases they are centered on the cortex of the diaphysis of the femur or tibia [1]. Within the femur, lesions are usually found proximally, most commonly within the neck and inter-trochanteric region [1]. It is known that location of osteoid osteomas in cancellous bone is rare and even rarer in intra-capsular locations [2]. However the exact incidence of juxta-articular osteoid osteomas in the femoral head is not known. In most cases, affected individuals complain of severe pain related to the lesion which is worse at night and relieved by ingestion of non-steroidal anti-inflammatory agents [3].

Plain radiographs demonstrate the nidus in 85% of cases. A total of 20% of cases may be intra-medullary and have less reactive sclerosis [4]. When intra-capsular in location, an osteoid osteoma may present with clinical features that mimic inflammatory synovitis and with atypical radiological findings such as lack of both sclerosis and periosteal reaction [5]. Magnetic resonance imaging (MRI) is less sensitive than computed tomography (CT) and allows detection of marrow edema and associated soft tissue edema; a nidus is identified in only 65% of cases with MRI. CT scanning improves detection of the nidus to more than 85% [6].

Surgery remains the standard treatment in cases where histology of the lesion is in doubt, neurovascular structures are within 1.5 cm, or in cases with repeated failure of any other minimally invasive ablative technique or percutaneous resection [7]. Successful surgical therapy occurs in 88% to 100% of cases. Primary radiofrequency ablation in a case series of over 200 patients has had a success rate of 76% to 100% [6]. In another series the primary and secondary success rates of this technique were 87% and 83%, respectively. Surgical resection and open curettage show comparable success rates, but are associated with higher complication rates [8].

### Case presentation

A 40-year-old Indian man from West Bengal presented to our facility with progressive left hip pain of insidious onset for a duration of five years. The pain had worsened in the six months prior to presentation, and was continuous, dull and aching in nature and relieved with analgesics. His clinical examination was unremarkable except for mild tenderness over the left hip anterior joint line. All hip movements were normal and pain free.

**Figure 1:**
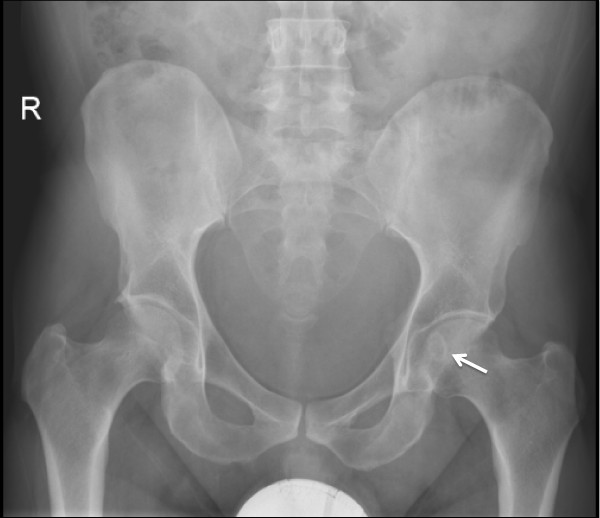
**Plain radiograph in anteroposterior view showing a well defined lytic lesion with a thin sclerotic rim located in the subarticular portion of the left femoral head (white arrow)**.

**Figure 2:**
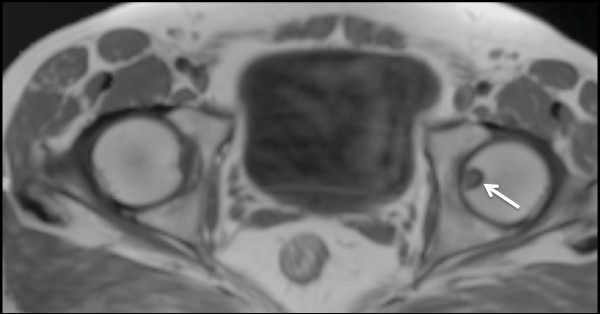
**T1-weighted axial MRI showing a corresponding hypointense lesion (white arrow)**.

**Figure 3:**
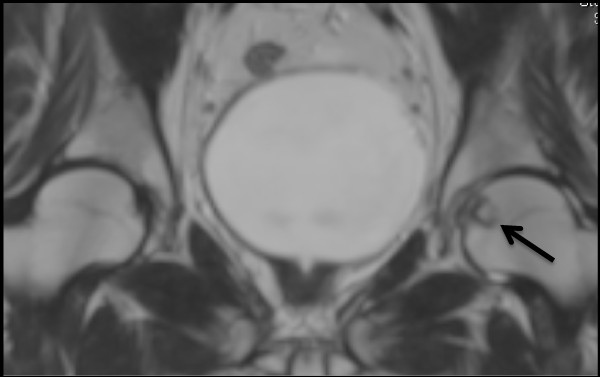
**T2-weighted coronal image showing hyperintense focus with a hypointense rim (black arrows)**.

**Figure 4:**
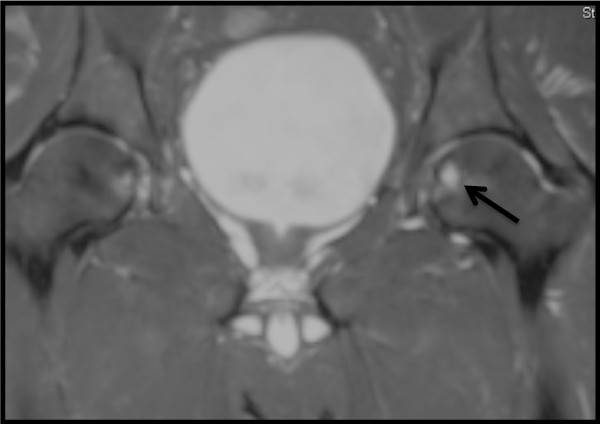
**T2 fat-suppressed images in coronal sections showing hyperintense focus with a hypointense rim (black arrows)**.

**Figure 5:**
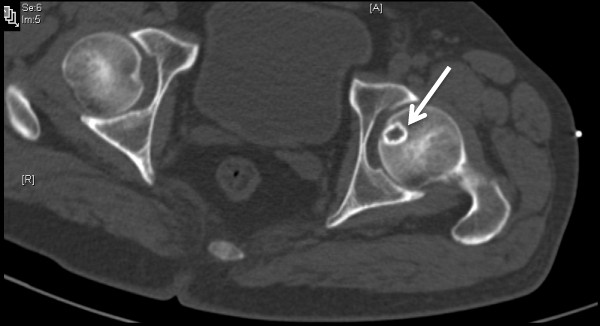
**Axial computed tomography section confirming a clearly defined lucent nidus with surrounding sclerotic rim (white arrow)**.

**Figure 6:**
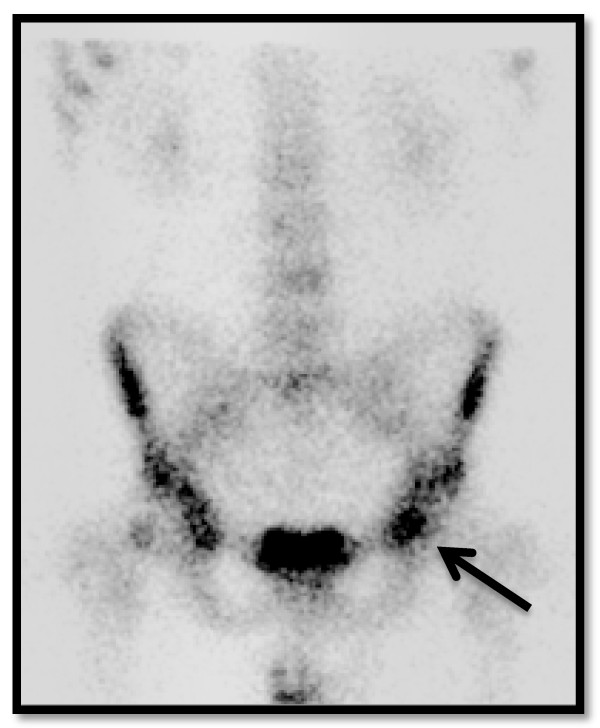
**Radionuclide bone scan demonstrating corresponding focal hot spot (black arrow)**.

Plain radiographs of the pelvis revealed a 15 × 11 mm, well defined lytic lesion with a thin sclerotic rim located in the subarticular portion of the left femoral head. Figure 1 shows a plain radiograph in anteroposterior view showing a well defined lytic lesion with a thin sclerotic rim located in the subarticular portion of the left femoral head (white arrow). On MRI, the lesion was hypointense on T1-weighted imaging and hyperintense with a hypointense rim on T2-weighted imaging. Figure 2 shows a T1-weighted axial MRI showing a corresponding hypointense lesion (white arrow). Figure 3 shows a T2-weighted coronal image showing hyperintense focus with a hypointense rim (black arrows). Figure 4 shows T2 fat-suppressed images in coronal sections showing hyperintense focus with a hypointense rim (black arrows). CT sections confirmed the above findings and revealed a distinct nidus measuring 11 × 10 mm. Figure 5 shows an axial CT section, confirming a clearly defined lucent nidus with surrounding sclerotic rim (white arrow). A radionuclide bone scan (Figure 6) revealed a focal hot spot at this site (black arrow).

**Figure 7:**
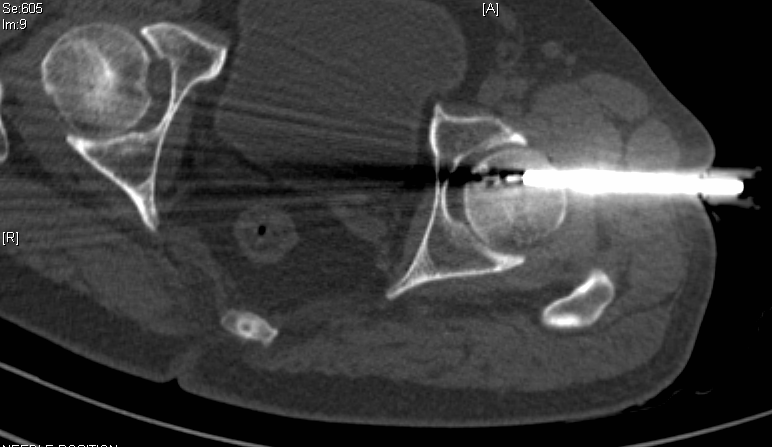
**Axial computed tomography section with radiofrequency ablation (RFA) needle placed within the drilled tract**.

**Figure 8:**
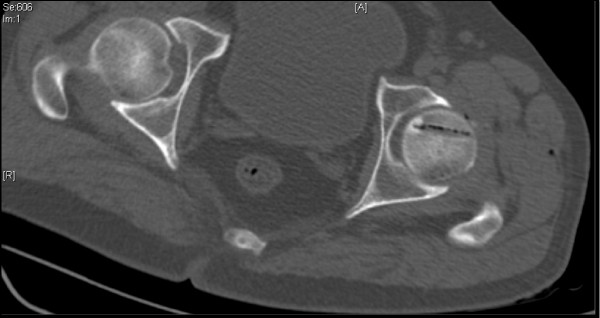
**Residual air pockets post radiofrequency ablation**.

**Figure 9:**
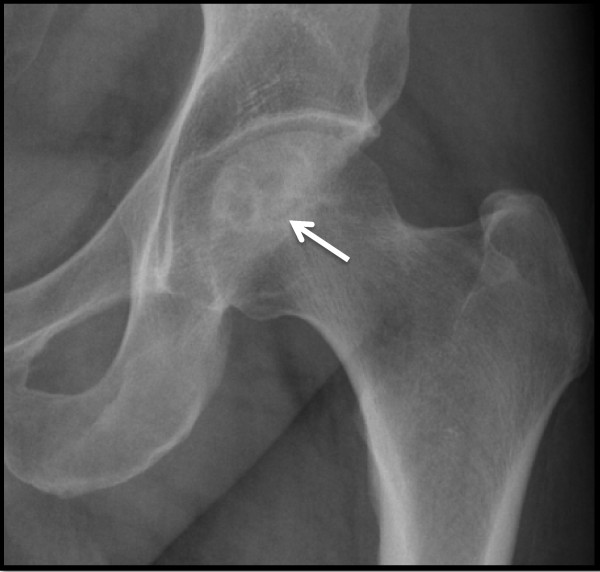
**Plain radiograph in anteroposterior view (white arrow) at review four months post procedure**.

**Figure 10:**
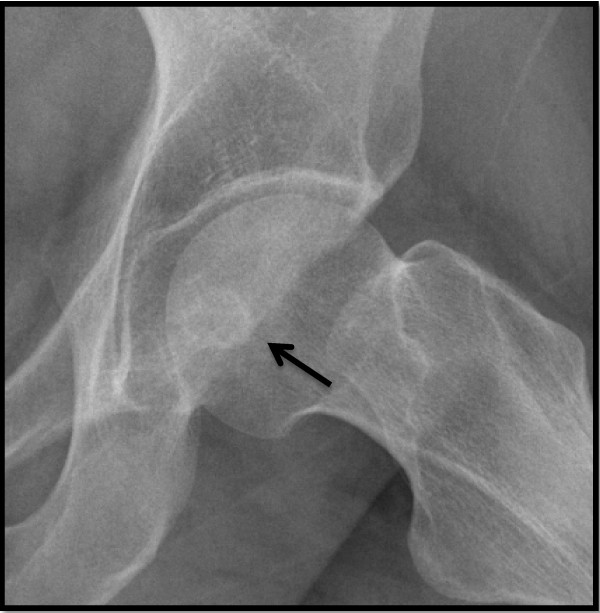
**Plain radiograph frog leg lateral views (black arrow) showing resolution of the lesion**.

Despite the uncharacteristic location, based on the imaging features a diagnosis of osteoid osteoma was made. After informed consent was obtained it was decided to perform a radiofrequency ablation. Under general anesthesia the nidus was localized with 3 mm CT sections and osseous access was established with a 4.5 mm drill. Figure 7 shows an axial CT section with radiofrequency ablation (RFA) needle placed within the drilled tract. After localization, the RFA needle (Starburst SDE, RITA Medical Solutions, Mountain View, CA, USA) was introduced through the drilled canal and tip placed in the nidus. Monopolar RFA was performed at a 90°C for a period of 5 minutes at 60 W. Figure 8 shows residual air pockets post radiofrequency ablation. The procedure was deemed successful as our patient was pain free within 24 hours of the procedure and remained so at follow-up. Figure 9 shows a plain radiograph in anteroposterior view (white arrow) at review 4 months post procedure. Figure 10 shows plain radiograph frog leg lateral views (black arrow) showing resolution of the lesion.

## Conclusion

RFA is an excellent alternative to surgical excision in the foveal region as it avoids the complications associated with surgical exposure of the femoral head, including injury to the capsular vessels and post-operative capsular laxity. It also avoids weakening of the femoral neck by large diameter drilling for surgical access and chondral or osteochondral damage from resection of the subchondral lesion. Furthermore, in this location there exists a potential risk of avascular necrosis owing to close proximity of the foveal artery in the ligamentum teres. The foveal artery is a branch of posterior division of the obturator artery, which becomes important to avoid avascular necrosis of the head of the femur when the blood supply from the medial and lateral circumflex arteries are disrupted.

In summary, the unusual finding in this index case is the relative absence of bone thickening, which could be due to the intra-capsular location. RFA is a better option than surgery in this location as it avoids injury to the articular margin, prevents capsular injury and reduces the risk of weakening the femoral neck. Injury to the foveal artery with the potential risk of avascular necrosis must be kept in mind when the lesion is close to the fovea of the femoral head.

## Consent

Written informed consent was obtained from the patient for publication of this case report and accompanying images. A copy of the written consent is available for review by the Editor-in-Chief of this journal.

## Competing interests

The authors declare that they have no competing interests.

## Authors' contributions

Image interpretation and RFA was performed by SKNK and KMM. KMM was a major contributor to writing the manuscript. KMJ was the orthopedic surgeon involved in acquiring osseous access with the drill. All authors read and approved the final manuscript.
